# Exopolysaccharide dispelled by calcium hydroxide with volatile vehicles related to bactericidal effect for root canal medication

**DOI:** 10.1590/1678-775720160014

**Published:** 2016

**Authors:** Lei Lei, Meiying Shao, Yan Yang, Mengying Mao, Yingming Yang, Tao Hu

**Affiliations:** 1 Sichuan University, West China Hospital of Stomatology, Department of Operative Dentistry and Endodontics, State Key Laboratory of Oral Diseases, Sichuan, China.; 2 Sichuan University, West China Hospital of Stomatology, Department of Preventive Dentistry, Sichuan, China.; 3 Sichuan University, College of Life Sciences, State Key Laboratory of Oral Diseases, Sichuan, China.; 4 The Forsyth Institute, Department of Microbiology, Cambridge, United States.

**Keywords:** Calcium hydroxide, Medications, Disinfection, Enterococcus faecalis, Exopolysaccharide

## Abstract

**Objective:**

*Enterococcus faecalis* is the dominant microbial species responsible for persistent apical periodontitis with ability to deeply penetrate into the dentin. Exopolysaccharides (EPS) contribute to the pathogenicity and antibiotic resistance of *E. faecalis*. Our aim was to investigate the antimicrobial activity of calcium hydroxide (CH), camphorated parachlorophenol (CMCP), and chlorhexidine (CHX) against *E. faecalis* in dentinal tubules.

**Material and Methods:**

Decoronated single-canal human teeth and semicylindrical dentin blocks were incubated with E. faecalis for 3 weeks. Samples were randomly assigned to six medication groups for 1 week (n=10 per group): CH + 40% glycerin-water solution (1:1, wt/vol); CMCP; 2% CHX; CH + CMCP (1:1, wt/vol); CH + CMCP (2:3, wt/vol); and saline. Bacterial samples were collected and assayed for colony-forming units. After dentin blocks were split longitudinally, confocal laser scanning microscopy was used to assess the proportion of viable bacteria and EPS production in dentin.

**Results:**

CMCP exhibited the best antimicrobial activity, while CH was the least sensitive against *E. faecalis* (p<0.05). CHX showed similar antimicrobial properties to CH + CMCP (1:1, wt/vol) (p>0.05). CH combined with CMCP inhibited EPS synthesis by *E. faecalis*, which sensitized biofilms to antibacterial substances. Moreover, increasing concentrations of CMCP decreased EPS matrix formation, which effectively sensitized biofilms to disinfection agents.

**Conclusion:**

The EPS matrix dispelled by CH paste with CMCP may be related to its bactericidal effect; the visualization and analysis of EPS formation and microbial colonization in dentin may be a useful approach to verify medicaments for antimicrobial therapy.

## INTRODUCTION

Microbiological sampling and examination of teeth with failed root canal treatments have shown that the microbial flora is predominantly comprised of gram-positive organisms. *Enterococcus faecalis* (*E. faecalis)* is considered a predominant organism that is frequently isolated from persistently infected root canal^18^. *In vivo* model, oral bacteria can penetrate up to 200 mm into dentinal tubules, which may make the bacteria resistant to antimicrobial agents^3^. The residual microbial flora located in inaccessible areas of the root canal anatomy is the main cause of persistent periapical infections. Because of anatomical complexities that cannot be reached by instruments, it is still difficult to clean root canals mechanically. Thus, intracanal medication has been proposed as a component of instrumentation and irrigation to eliminate bacteria along with their by-products^15^.

Calcium hydroxide (CH) paste is one of the most commonly used intracanal medicaments. When dissolved in water, the CH paste dissociates into hydroxide and calcium ions; as a result, the antimicrobial action of this medication depends on the presence of hydroxide ions in the solution^5^. Despite its excellent properties, the buffering action of dentin can neutralize the antimicrobial activity of CH at deeper layers of dentinal tubules, and *E. faecalis* resistance to this medicament has consequently been demonstrated^19^. The camphorated parachlorophenol (CMCP) solution contains chemical ingredients, such as chlorophenol, and the antimicrobial actions of CMCP products are related to its ability to destroy the bacterial membrane through protein and lipid binding. The association of CH paste and CMCP forms calcium p-chlorophenolate, which increases the pH of dentin, allowing the liberation of hydroxyl ions for an extended time^17^. The addition of camphorated parachlorophenol (CMCP) to CH paste was more effective to eliminate the microorganisms in infected root canals, based on the method of bacteriological sampling in root canals using paper points^16^. The association of CH with CMCP may enhance the diffusion of CH, while maintaining a relatively high pH value. Furthermore, the addition of CH paste has been shown to decrease the toxicity of CMCP in periapical tissues via prolonged action of the calcium hydroxide-based paste^9^. When the CH paste combined with CMCP was used as an intracanal medication, 74% of the successes were categorized as cases of two-visit treatment^24^. However, this methodology is limited, since the microorganisms located inside the dentin were not collected.

The chlorhexidine (CHX) is positively charged and has been reported to act by electrostatic interactions with the negatively charged bacterial wall. This procedure alters the cell osmotic equilibrium and results in cell death, which contributes to the antimicrobial effect of CHX^6^. Two percent CHX gel is used as an intracanal dressing, in which it is effective against microorganisms in infected root canals^16^. The challenge of eliminating all bacteria in root canals may be partly attributed to their invasion into dentinal tubules, where the organisms are protected from medicaments. The addition of CMCP to CH paste may enhance the diffusion of CH and significantly decrease the toxicity of CMCP in periapical tissues^9^. Thus, we sought to examine the efficacy of CH paste, CMCP, and CHX gel to eliminate bacteria within dentinal tubules.

*E. faecalis* becomes more resistant in the root canals because of its ability to adapt to harsh environmental changes and starvation^13^. Exopolysaccharides (EPS) are the crucial components of the protective shelter in oral biofilms^2^. Consequently, the dominant mechanism involved in biofilm resistance to antimicrobial therapy may be the capability of EPS to provide mechanical stability and drug tolerance. Additionally, treating *E. faecalis* biofilms with dextranase effectively sensitized bacteria to a 2% CHX solution, which highlighted the hypothesis that dispelling EPS provides a new target for antimicrobial therapies^12^. However, little is known about the effects of medicaments on EPS synthesis and *E. faecalis* survival in dentinal tubules.

Recently, a dentin infection model was generated to establish standard and deep penetration of bacteria into dentinal tubules^1,14^. This visual strategy efficiently assessed the efficacy of endodontic medication against *E. faecalis* in dentin^20^. In addition, the contamination protocols and the method for the microbial infection of the dentine had been modified in the *in vitro* model^1^ and investigated in intra-oral appliances^3^. As *E. faecalis* play a central role in endodontic infections, with EPS likely contributing to its pathogenicity and antibiotic resistance, this study sought to investigate the antimicrobial efficacy of current medicaments against *E. faecalis* in infected canals and the role of EPS in this process.

## MATERIAL AND METHODS

### Sample preparation

**Figure 1:**
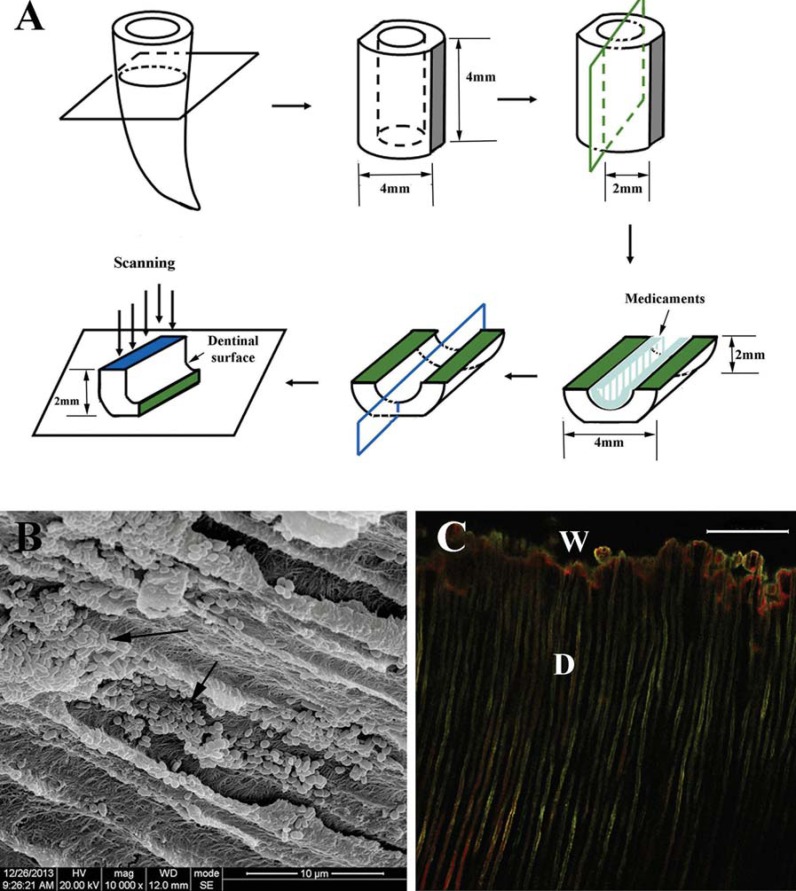
Schematic drawings of sample preparation and medication. (A) Bacterial colonization in dentinal tubules; (B) Successful bacterial colonization in dentinal tubules was shown by scanning eletron microscopy observation (black arrow); (C) Two-dimensional images of CLSM showed mixtures of single cells inside the dentin. W, root canal wall; D, dentinal tubules; green, viable bacteria (SYTO 9); red, dead bacteria (PI); Scale bar, 100 μm

One hundred and twenty extracted single-canal human teeth were extracted from adults (18–30 years old) for orthodontic reasons and were kept in a 0.01% NaOCl solution prior to use. Each participant was provided with an informed consent form approved by the Ethics Committee of West China Hospital of Stomatology, Sichuan University. The teeth were prepared using a Protaper system to F3 file^7^. The debris and smear layer were removed by immersion in an ultrasonic bath containing 5.25% NaOCl for 5 minutes, then were submerged in 17% EDTA for 5 minutes. Each root was decoronated to obtain roots measuring 12 mm in length and sterilized before use. One hundred and twenty semicylindrical dentin halves were shaped as previously reported^14^. Dentin blocks with a length of 4 mm were horizontally sectioned from each tooth at 1 mm below the cementoenamel junction. Root canal blocks were enlarged to a size of Gates Glidden drill #6 (Tulsa Dentsply, Tulsa, OK, USA). Each cylindrical dentin block was fractured by prepared grooves into two semicylindrical halves. The outer surfaces of the dentin blocks were ground to achieve a standard thickness of 2 mm (Figure 1A). The smear layer of the specimen was removed by immersion in 5.25% NaOCl, then 17% EDTA for 5 minutes each in an ultrasonic bath. The outer sides of the roots and dentin blocks were sealed by nail varnish to close the open dentinal tubules. The average colony-forming unit (CFU) counts and the standard deviations were defined according to our preliminary work. Then, an assumption was made that the statistical significance level is typically 5% (α=0.05) and adequate power is widely accepted as 0.9 (90%). The hypothesized means and standard deviations were calculated. The sample size, estimated by PASS (Power Analysis and Sample Size) version 13.0 (NCSS, Kaysville, UT, USA), was 7 samples *per* individual group; thus, 10 samples were included in each group to maintain an adequate sample size.

### Dentin infection with *E. faecalis*

The *E. faecalis* standard strain ATCC 29212 was aerobically incubated at 37°C for 24 hours on brain heart infusion (BHI; Difco, Sparks, MD, USA) agar plates, then a pure single colony was suspended in BHI medium. The cell suspension was monitored by CFU assay, and bacterial cultures were adjusted to 1×10^7^ CFU/mL; the optical density (OD) of the cell culture at 600 nm was adjusted to OD of 0.1^7^. One hundred and twenty single-canal samples were incubated with 5×10^6^ CFU in 500 μL of *E. faecalis* for 3 weeks. Fresh BHI medium was replaced every 48 hours. One hundred and twenty semicylindrical dentin blocks were placed in 6-well polystyrene cell culture plates with dentinal surfaces facing upwards^20^. Dentin specimens were incubated with the bacterial suspension in BHI medium with 1.0% sucrose for 3 weeks and fresh BHI medium was replaced every 48 hours.

### Disinfection of dentin

After bacterial infection, two additional specimens from each group were longitudinally split for scanning electron microscopy (SEM; Inspect F50, FEI, Hillsboro, OR, USA) to confirm bacterial colonization. Dentin blocks were longitudinally split through vertically prepared grooves into two halves, and the fresh surface of fractured visible dentin was washed twice with PBS and fixed with 2.5% glutaraldehyde for 8 hours. The samples were then serial dehydrated through ethanol solutions (30%, 50%, 70%, 95%, then 100%), subjected to critical-point drying with liquid CO_2_, and coated with gold for imaging. Three randomly selected areas from each sample were imaged by SEM. The bacteria-infected specimens were randomly divided into six groups as follows: Group A: CH + 40% glycerin-water solution (at a powder to liquid ratio of 1:1, wt./vol.; Zhangjiang Biomaterials Co. Ltd, Shanghai, China); Group B: CMCP (30 mg/mL camphor and 15 mg/mL phenol; Longly Biomaterials Co. Ltd, Wuhan, China) paper points; Group C: 2% CHX gel (Nanyue pharmaceutical Co. Ltd, Shenzhen, China); Group D: CH + CMCP (1:1, wt./vol.); Group E: CH + CMCP (2:3, wt./vol.); and Group F: saline. Fifty microliters of each medicament was introduced into root canals using a spiral conveyer or placed on the dentinal surface of each dentin halve for 1 week (Figure 1A).

### Microbiological sampling analysis

Bacterial samples were taken before the administration of the medication (S1). The specimens were washed three times for 30 seconds each in ultrasonic bath using sterile saline^5^. Root canal walls were gently filed in 50 μL of saline and a post-medication sample (S2) was obtained with one 30# paper point^25^. The microbiological samples were serially diluted in saline (10^−1^ to 10^−7^ dilutions) and each dilution was plated onto BHI agar plates and aerobically incubated at 37°C for 48 hours. The number of CFU per square millimeter was calculated^12^.

### CLSM analysis

After removal of the medicaments in an ultrasonic bath, the dentin blocks were longitudinally split through vertically prepared grooves into two halves to expose the fresh surface of fractured visible dentin (Figure 1A): one half was processed and stained with 50 μL of LIVE/DEAD BacLight^TM^ Bacterial Viability Kit reagent (Molecular Probes Inc., Eugene, OR, USA) according to the manufacturer's instructions to assess the proportion of vital bacteria. Briefly, live and dead cells were differentiated by staining with SYTO9 dye (green fluorescence) and propidium iodide dye (red fluorescence), respectively, at a concentration of 1 mg/mL for 15 minutes in the dark at room temperature. The samples were analyzed with a CLSM (Type TSP SP2; Leica, Solms, Germany) using 480/500 nm lasers for SYTO 9 stain and 605/635 nm lasers for propidium iodide^12^. The border of the freshly fractured dentin surface was first located with a microscope, and three randomly selected fields of view were scanned. The volume ratio of green fluorescence to green-and-red fluorescence indicated the portion of live cells for each disinfecting agent.

For EPS matrix imaging, the other half of the semicylindrical dentin block in each group was examined via fluorescent labeling of EPS and bacterial cells. The EPS matrix was stained with Alexa Fluor647-labeled dextran conjugate (Molecular Probes Inc.) and bacterial cells were labeled with SYTO9^28^. Particularly, the Alexa Fluor 647-labeled dextran was applied before the dentin infection. The Alexa Fluor 647 dye was added to the prepared bacterial suspension adjusted to a concentration of 1 mg/mL, which was subsequently incubated with the dentin samples for 3 weeks^11^. The spectra were taken with a wavelength range of 610/750 nm for the Alexa Fluor 647-labeled dextran conjugate (red fluorescence) and 480/500 nm lasers for SYTO 9 stain (green fluorescence) at a resolution of 512×512 pixels^28^. The fluorescence intensity quantification was analyzed using Imaris 7.0 software (Bitplane, Zurich, Switzerland). The volume ratio of red fluorescence to green fluorescence in the three-dimensional reconstructions indicated the EPS matrix to bacterial cells ratio. Three-dimensional reconstruction of the biofilms was performed, and this procedure was repeated in triplicate for three randomly selected views for each specimen.

### Statistical analysis

The values of CFUs/mL were pretreated with logarithmic transformation. The Shapiro-Wilk test demonstrated whether the data were normally distributed, and Bartlett's test was used to assess the homogeneity of variances. For parametric testing, Fisher's tests and one-way ANOVA were used to compare the data using SPSS 16.0 (SPSS Inc, Chicago, IL, USA), and statistical significance was set at p=0.05.

## RESULTS

### Antibacterial action of medicaments against *E. faecalis* in infected root canals

**Figure 2:**
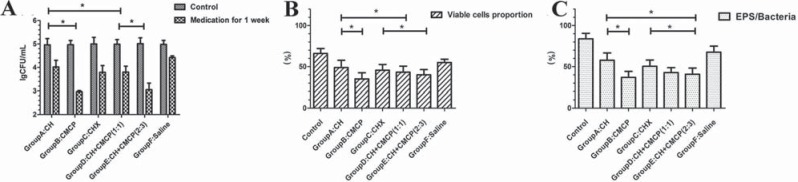
Antimicrobial effect of different medicament regimens on *E. faecalis* in infected canals. *Significant differences among the medicament groups (p<0.05, n=10). (A) Number of CFUs after the administration of intracanal medication [ ± s; n=10, lg (CFU/mL)]. The results showed that the decrease in CFUs in Group A, Group B, Group C, Group D, Group E, and Group F was 0.94 logunits, 2.01 logunits, 1.21 logunits, 1.19 logunits, 1.96 logunits, and 0.56 logunits, respectively; (B) Percentage (%) of viable *E. faecalis* cells that penetrated into the dentin after the administration of medication ( ± s, n=10). The mean proportions of viable populations were 48.9% in Group A and 35.0% in Group B; (C) Volume ratio of EPS matrix to bacteria in the dentin after medication. Group A had the highest ratio; Group B exhibited the lowest EPS matrix/ bacteria ratio (p<0.05, n=10); Group E showed the second-lowest ratio

In general, CFU counts significantly decreased after intracanal medication, indicating antimicrobial activity of the medicaments (Figure 2A). CMCP (30 mg/mL camphor and 15 mg/mL phenol) exhibited the best antimicrobial activity, while CH was the least sensitive against *E. faecalis* (p<0.05). The average CFU counts in the CMCP group was the lowest (p<0.05, n=10). The CH + 40% glycerin-water solution CMCP (2:3, wt/vol) was more effective than the 2% CHX and CH + 40% glycerin-water solution CMCP (1:1, wt/vol) (p<0.05, n=10), and there were no significant differences between the latter two groups (p>0.05, n=10).

### Antibacterial activity of medicaments on *E. faecalis* and EPS synthesis in dentinal tubules

**Figure 3:**
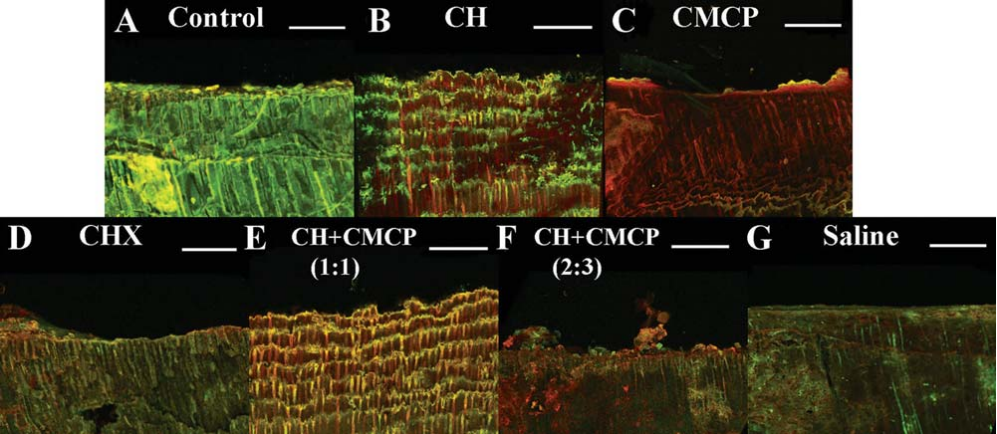
Three-dimensional reconstruction of CLSM images of *E. faecalis* colonization in dentinal tubules. (A) The sample that was taken before the dentin specimen was treated and used as control. *E. faecalis* in dentin were treated with (B) CH paste, (C) CMCP paper points, (D) 2% CHX gel, (E) CH + CMCP paste (1:1, wt/vol), (F) CH + CMCP (2:3, wt/vol), and (G) saline solution. Green, viable bacteria (SYTO 9); red, dead bacteria (PI); Scale bars, 50 μm

**Figure 4:**
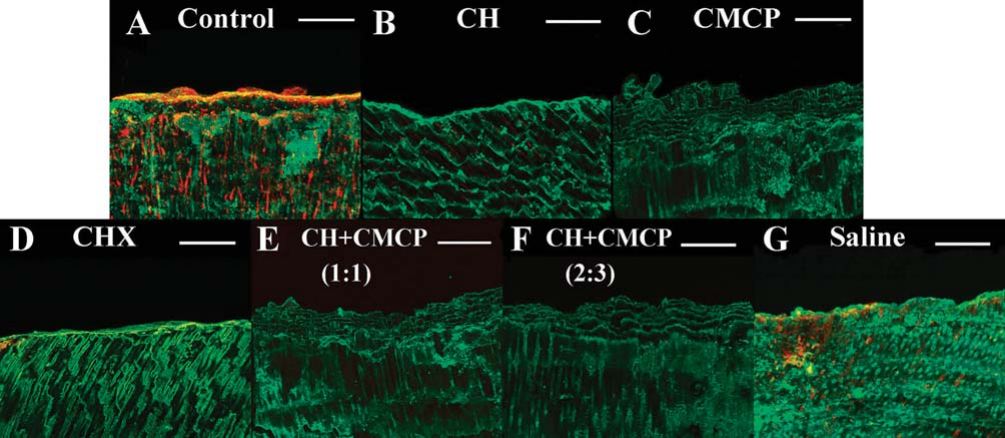
Three-dimensional reconstruction of CLSM images of *E. faecalis* colonization and EPS matrix in dentinal tubules. (A) The sample that was taken before the dentin specimen was treated and used as control. *E. faecalis* in dentin were treated with (B) CH paste, (C) CMCP paper points, (D) 2% CHX gel, (E) CH + CMCP paste (1:1, wt/vol), (F) CH + CMCP (2:3, wt/vol), and (G) saline solution. Green, total bacteria (SYTO 9); red, EPS (Alexa Fluor 647); Scale bars, 50 μm

Successful bacterial colonization in dentinal tubules was observed by SEM (Figure 1B), and a dense penetration of *E. faecalis* was observed in the dentin up to 300 μm (Figure 1C). CLSM showed mixtures of single cells or abundant clusters of cells and EPS matrix inside the dentin (Figures 3A and 4A). After medication, the proportion of viable bacteria ranged from 30% to 60%, depending on the medicaments (Figure 2B), and the lowest percentage of live bacteria was detected in the CMCP group (p<0.05, n=10). The proportion of viable cells in the CH + 40% glycerin-water solution CMCP (2:3, wt/vol) group (40.0%) was lower than the 2% CHX (45.6%) and CH + 40% glycerin-water solution CMCP (1:1, wt/vol) (45.2%) groups (p<0.05, n=10). To clarify the relationship between microbial colonization and EPS synthesis, the ratio of EPS matrix to bacterial cells was calculated (Figure 2C). After administration of medication (Figure 4), the CMCP group exhibited the lowest EPS/bacterial cell ratio, whereas the CH group exhibited the highest ratio (p<0.05, n=10).

## DISCUSSION

*E. faecalis* was shown to produce a dense infection into dentinal tubules, reaching up to 300 μm at three weeks post-infection. In the present study, either CMCP or CH combined with CMCP demonstrated excellent antibacterial activity against *E. faecalis*, similar to previous reports^25^. Our results further demonstrated that inhibition of EPS synthesis by *E. faecalis* sensitized the biofilms to medicament-induced killing, suggesting that the EPS matrix plays a crucial structural role in *E. faecalis* survival.

### Addition of CMCP enhanced the antibacterial action of CH paste against *E. faecalis* in infected canals

The effectiveness of CH is linked to the diffusion of hydroxyl ions through the dentinal tubules and accessory canals into areas that are inaccessible to instruments. When dissolved in water, the CH paste dissociates into hydroxide and calcium ions; as a result, the antimicrobial action of this medication depends on the concentration of hydroxide ions in the solution^5^. The antibacterial activity of CH paste has been previously questioned because of the buffering potential of the dentin, which decreases the pH effect^5,19^. Previously, the influence on dissociation of CH in aqueous and nonaqueous solutions has been investigated, and the conductivity of CH in pure glycerin or propylene glycol was measured^22^. In this study, the conductivity of CH in pure glycerin was negligible and the conductivity value of CH in water was 7.3±3 mS/cm. These results indicate that high concentrations of glycerin reduce conductivity^22^. In the present study, the CH paste mixed with a 40% solution of glycerin had some antimicrobial action, but was inferior to the CH paste mixed with CMCP solution. It is possible that higher concentrations of glycerin may impede the effectiveness of CH as an intracanal dressing. On the other hand, *E. faecalis* is able to endure alkaline stress at a pH value of 11.1^13^. Therefore, this combination of CH paste and CMCP solution was superior to the CH paste alone in eliminating the microorganisms in root canals.

In addition, the application of CH paste is unable to achieve a pH value greater than 10 within the dentinal tubules because of the dentin buffering effects^10^. Previous studies report that *E. faecalis* can form biofilms and colonize dentinal tubules under alkaline stress conditions *in vitro* ^20^. As a result, CH combined with CMCP has been suggested to provide a broader antibacterial spectrum and more rapid penetration than inert vehicles^25^. In the present study, CH combined with CMCP exhibited a more pronounced antibacterial effect than CH alone, indicating that this association may compensate for the weaknesses of CH as previously proposed. Ideally, an intracanal medication should occupy the root canal space reaching into the infected dentinal tubules to destroy the microorganisms. As phenolic compounds have a low surface tension, the combination of CH and CMCP may allow for an easier flow of the medicament into the dentinal tubules and also appears to be less toxic than CMCP alone^9^.

The antibacterial effect of CH pastes has been attributed to dissociation of hydroxyl ions. However, the buffering action of dentin can neutralize the antimicrobial activity of CH at deeper layers of dentinal tubules^5^. In addition, CH pastes associated with CMCP allow a controlled liberation of calcium and hydroxyl ions. This association forms calcium p-chlorophenolate, which increases the liberation of hydroxyl ions for a longer period^17^. That kind of reaction between those two medications may be less toxic than CMCP alone. On the other hand, CH combined with CMCP composes a lesser amount of CMCP than when is used alone in the present study. When incorporated with the CH paste, reduced concentration of CMCP would also contribute to less the toxicity of CMCP as the root canal medicament. The antimicrobial effect of CMCP products is often described as its ability to destroy the bacterial membrane by binding on its proteins and lipids. As a result, CMCP solution is controversial when used as medication because of its possible toxicity caused by ingredients such as chlorophenol^9^. Because of the fast diffusion of CMCP solution through the dentinal tubules, adverse cytotoxic reactions were found when there was liquid extrusion in the periapical tissues. However, the denaturing effect of CH paste on periapical tissues may prevent further tissue penetration by CMCP^25^. From this point of view, it is supposed that this association of CMCP + CH paste would be advantageous by slow release of CMCP from the paste. In addition, the combination of CH and CMCP could increase the pH of dentin and help to maintain the alkaline environment^17^. The high pH may promote a superficial denaturing effect of CH paste in which this area may act as a physical barrier to a deeper diffusion of chlorophenol into the periapical tissues^9^. Conversely, this association may increase the pH of dentin, promoting antimicrobial action^9,25^. The current results also indicate that increasing the concentration of CMCP enhances its antibacterial efficacy.

The antimicrobial effect of chlorhexidine (CHX) is reported to act by electrostatic interactions, in which CHX is positively charged and the bacterial wall is negatively charged. This interaction alters the cell osmotic equilibrium and allows bacterial cytoplasm coagulation, resulting in cell death^6^. Two percent CHX gel is commonly used as a chemical auxiliary in endodontic therapy, which shows its effectiveness against microorganisms present in infected root canals^16^. The association of CH and 2% CHX has been used as an intracanal dressing because of its enhanced antimicrobial action against endodontic pathogens^8^. It has been found that CHX may induce reactive oxygen species (ROS) production in the alkaline environment with a biphasic response, which are possibly involved in the killing of root canal microorganisms^29^. The CH pastes have the ability to induce hard tissue formation and also mediate the neutralization of lipopolysaccharide (LPS)^15^. As a result, it is supposed that this association of CHX gel + CH paste would be advantageous compared with CH paste alone, because the substantive effect of CHX sustains the antimicrobial activity after being removed from the root canal systems^23^. However, the association of CHX with CH may fail to improve the antibacterial efficacy of CHX gel^26^. In the present study, it was observed that the antimicrobial activity of CH paste was inferior to the CHX gel alone. On the other hand, the present investigation also demonstrated that the antimicrobial effect of CHX was inferior to that of CH combined with CMCP (2:3, wt./vol.), which was related to the poor penetration into the matrix-encased structure of the biofilm. CHX appears to be ineffective in dissolving glycosidic bonds in the biofilm matrix^26^. Utilization of CH with CMCP may provide a better therapeutic strategy, since CHX may interact with residual sodium hypochlorite, inducing tooth discoloration. Furthermore, CHX may interfere with the sealing ability of root canal fillings^21^.

### Dispelling the EPS Matrix sensitized *E. faecalis* in dentinal tubules to medicaments

Microbial flora colonization is considered to be the critical first step in biofilm formation and dentinal tubule invasion^20^. EPS are composed of microbial dextran derived from or found on the cell walls of *E. faecalis*. In addition to initiating the attachment and colonization of biotic surfaces^4^ by planktonic cells, EPS also act as an extracellular digestive system by trapping enzymes that surround bacterial cells, which reduces physical contact with antimicrobial agents^2^. For the survival of *E. faecalis*, it is presumed that bacteria may remain alive, but nonculturable (VBNC), in nutrient poor environments^27^. Thus, the bacteria left in dentinal tubules after instrumentation and irrigation may grow again in the presence of an EPS matrix. Furthermore, dextran, a class of extracellular-formed glucans produced by bacteria, has been found in the matrix of *E. faecalis* biofilms, implicating dextran in the development of microbial communities^12^.

Previous reports demonstrated that the Alexa Fluor 647-labeled dextran generated a fluorescence resonance energy-transfer (FRET) reaction, in which the dextran chain was displaced, resulting in a change in fluorescence^11^. In addition, we slightly modified the standard dentin infection model to achieve a better understanding of the remaining bacterial distribution and of EPS organization in dentinal tubules. In the present study, autofluorescence in noninfected dentinal tubules could be visualized after staining, which is consistent with previous investigation^14^. Considering that all dentin blocks were prepared to achieve the standard size (4×2×2 mm), dentinal specimen sampling maintained consistent baselines for CLSM observation and could ensure valid comparisons to be made between the experimental medication groups. After dentin blocks were longitudinally split, CLSM observations were conducted to assess the proportion of viable bacteria and EPS production in dentin, which, thus, was established in relevant publications^14^. In addition, dentinal blocks incubated with saline as control were also investigated and the control group could also be compared as the baseline of the other medication groups (Figures 3 and 4).

The labeling technique allowed the visualization of VBNC cells and the quantitative analysis of the EPS matrix. In the present study, the visual strategy has been used to assess not only the percentage of vital bacteria, but also EPS matrix formation in the dentinal tubules. EPS production declined with a concomitant decrease in the proportion of live cells (Figure 2B-C), suggesting that reduced EPS production enhances the anti-biofilm activity of medicaments. Particularly, *E. faecalis* utilizes the residual EPS matrix in dentinal tubules after treatment with saline under starvation conditions (Figure 4G). As a result, the presence of EPS in deeper areas may create a chemical barrier against antibacterial agents^2,25^. Thus we propose that the increased resistance of *E. faecalis* in the dentinal tubules may be attributed to the EPS matrix. In the present study, the combination of CH and CMCP inhibited EPS synthesis and negatively affected bacteria in the dentin. The degradation of the EPS matrix facilitated the penetration of CMCP, which sensitized the bacteria present in dentinal tubules to antibacterial agents. Clearly, the quantitative analysis of EPS and microbial colonization would advance our understanding of biofilm structure and accelerate the development of antimicrobial agents. This study sought to investigate the role of EPS matrix involved in antimicrobial efficacy of current medicaments in infected canal conditions. In our opinion, the EPS matrix dispelling by root canal medication, which would be related to bactericidal effect of the disinfection agents. Further investigations should be carried out to clarify the role of CMCP in EPS matrix metabolism and to evaluate the viability of residual bacteria after root canal fillings.

## CONCLUSION

In summary, this study demonstrated that CMCP enhanced the antibacterial action of CH paste against *E. faecalis* and inhibited EPS synthesis in the dentin. Moreover, increasing concentrations of CMCP sensitized biofilms more effectively to disinfection agents. These two medicaments may additively act toward the elimination of *E. faecalis* and degradation of the EPS matrix, facilitating the modification of the structure of the biofilm. The antimicrobial activity of CHX gel was superior to the CH paste alone and was similar to CH combined with CMCP (1:1, wt./vol.). The labeling visualization and the quantitative analysis demonstrated that EPS matrix declined with a concomitant decrease in the proportion of live cells, suggesting that reduced EPS production may enhance the antimicrobial activity. The EPS matrix dispelled by CH paste with volatile vehicles may be related to its bactericidal effect for infected root canal medication. The visualization and analysis of EPS formation and microbial colonization in dentinal tubules may be a useful approach to verify antimicrobial components using an *in vitro* model.
